# Adrenal function testing in patients with septic shock

**DOI:** 10.1186/cc5077

**Published:** 2006-10-25

**Authors:** Diamantino Ribeiro Salgado, Juan Carlos Rosso Verdeal, José Rodolfo Rocco

**Affiliations:** 1 Intensive Care Unit, Barra Dor Hospital, Avenida Ayrton Senna 2541, Barra da Tijuca, Rio de Janeiro CEP 22775-001, Brazil; 2 Intensive Care Unit, Clementino Fraga Filho University Hospital, Federal University of Rio de Janeiro, Rio de Janeiro, Brazil

## Abstract

**Introduction:**

Adrenal failure (AF) is associated with increased mortality in septic patients. Nonetheless, there is no agreement regarding the best diagnostic criteria for AF. We compared the diagnosis of AF considering different baseline total cortisol cutoff values and Δmax values after low (1 μg) and high (249 μg) doses of corticotropin, we analyzed the impact of serum albumin on AF identification and we correlated laboratorial AF with norepinephrine removal.

**Methods:**

A prospective noninterventional study was performed in an intensive care unit from May 2002 to May 2005, including septic shock patients over 18 years old without previous steroid usage. After measurement of serum albumin and baseline total cortisol, the patients were sequentially submitted to 1 μg and 249 μg corticotropin tests with a 60-minute interval between doses. Post-stimuli cortisol levels were drawn 60 minutes after each test (cortisol 60 and cortisol 120). The cortisol 60 and cortisol 120 values minus baseline were called Δmax^1 ^and Δmax^249^, respectively. Adrenal failure was defined as Δmax^249 ^≤ 9 μg/dl or baseline cortisol ≤ 10 μg/dl. Other baseline cortisol cutoff values referred to as AF in other studies (≤15, ≤20, ≤25 and ≤34 μg/dl) were compared with Δmax^249 ^≤ 9 μg/dl and serum albumin influence. Norepinephrine removal was compared with the baseline cortisol values and Δmax^249 ^values.

**Results:**

We enrolled 102 patients (43 male). AF was diagnosed in 22.5% (23/102). Patients with albumin ≤2.5 g/dl presented a lower baseline total cortisol level (15.5 μg/dl vs 22.4 μg/dl, *P *= 0.04) and a higher frequency of baseline cortisol ≤25 μg/dl (84% vs 58.3%, *P *= 0.05) than those with albumin > 2.5 g/dl. The Δmax^249 ^levels and Δmax^249 ^≤ 9, however, were not affected by serum albumin (14.5 μg/dl vs 18.8 μg/dl, *P *= 0.48 and 24% vs 25%, *P *= 1.0). Baseline cortisol ≤ 23.6 μg/dl was the most accurate diagnostic threshold to determine norepinephrine removal according to the receiver operating characteristic curve.

**Conclusion:**

AF was identified in 22.5% of the studied population. Since Δmax^249 ^≤ 9 μg/dl results were not affected by serum albumin and since the baseline serum total cortisol varied directly with albumin levels, we propose that Δmax^249 ^≤ 9 μg/dl, which means Δmax after high corticotropin dose may be a better option for AF diagnosis whenever measurement of free cortisol is not available. Baseline cortisol ≤23.6 μg/dl was the best value for predicting norepinephrine removal in patients without corticosteroid treatment.

## Introduction

Septic shock and multiple organ failure are the most frequent causes of death in medical intensive care units (ICUs), and were responsible for approximately 750,000 admissions and 210,000 deaths throughout 1995 in the USA [1]. Although the mortality rate has been declining, the incidence of sepsis has increased, resulting in almost three times the number of sepsis-related deaths in the USA between 1979 and 2000 [2]. Furthermore, patients who survive sepsis have a worse long-term health-related quality of life [3,4].

A normally functioning hypothalamic–pituitary–adrenal axis is essential to organ homeostasis during acute illness, especially in severe infections. Patients with adrenal failure (AF) and septic shock are known to present high mortality rates [5]. Additionally, stress doses of hydrocortisone have been shown to reduce septic-shock-related mortality [6-8], mainly in patients with an impaired response to the corticotropin test [9,10].

The incidence of AF may vary widely, from 0% to 77%, depending on the studied population and the criteria used for the diagnosis [11]. Although chronic AF (Addison disease) is rare and relatively simple to identify, the acute presentation is not straightforward and is usually unrecognized. A high suspicion index is necessary since the classical clinical and laboratorial findings, such as hypotension, fever, abdominal pain, hypoglycemia, electrolyte abnormalities and eosinophilia, are easily mistaken for the critically ill condition.

The failure of serum cortisol to rise above 18 or 20 μg/dl at 30 or 60 minutes after administration on 250 μg corticotropin is classically considered diagnostic for adrenal failure in the absence of acute illness [12]. There is, however, no consensus on the best diagnostic criteria for AF in acutely ill patients. Some authors recommend the use of baseline cortisol, justifying that acute stress such as severe infections, surgery, shock and hypoxemia are enough stimuli to the adrenal glands [11,13,14]. Many cortisol cutoff values have been proposed, ranging from 10 to 34 μg/dl, but most researchers consider a baseline cortisol lower than 15 μg/dl to better identify patients presenting clinical features of corticosteroid insufficiency or those who would benefit from corticosteroid replacement [15,16]. Conversely, other authors consider that it is possible, by performing a low-dose (1 μg) [10] or mainly a high-dose (250 μg) corticotropin test [9,17], to identify patients with relative adrenal insufficiency when the difference between the highest serum cortisol value and the baseline cortisol value (Δmax) is lower than 9 μg/dl [17,18]. Some of these authors suggest these adrenal insufficiency patients could benefit from corticosteroid treatment [9,10,17]. Additionally, because more than 90% of circulating cortisol in human serum is bound to proteins (corticosteroid binding protein and albumin), either the baseline cortisol level or the Δmax value may be influenced by serum protein levels, and alterations in the binding proteins could affect the measured concentration of total serum cortisol [19-21].

The main aim of the present study was to compare the diagnosis of adrenal failure considering different baseline cortisol cutoff values and the Δmax value after a low dose (1 μg) and a high dose (249 μg) of corticotropin. Secondarily, we analyzed the impact of serum albumin on AF identification and we correlated the laboratorial diagnosis of AF with norepinephrine removal in patients with septic shock.

## Patients and methods

### Patients

This prospective and noninterventional study was carried out in an ICU of a tertiary private hospital from May 2002 to May 2005. The Institutional Ethics Committee waived the need for informed consent as the test is routinely performed in the unit.

We included patients over 18 years old admitted to the ICU with septic shock, defined by the American College of Chest Physicians/Society of Critical Care Medicine Conference Consensus Committee [22] as the presence of two or more of the following conditions as a result of infection: fever (temperature >38°C) or hypothermia (temperature <36°C); tachycardia (heart rate >90 beats/minute), tachypnoea (respiratory rate >20 breaths/minute) or hyperventilation (partial pressure of arterial blood CO_2 _<32 mmHg); altered white blood cell count (>12,000 cells/mm^3 ^or <4,000 cells/mm^3 ^or >10% band forms); and hypotension (mean arterial blood pressure <65 mmHg) with vasopressor drug requirement (norepinephrine at any dose) despite adequate fluid resuscitation.

Exclusion criteria were corticosteroid usage in the past year or its requirement as part of the treatment in the present admission, administration of etomidate, end-stage cancer, AIDS, chronic renal failure requiring hemodialysis support, cirrhosis (Child–Pugh class C), pregnancy, or limitation of lifecare support.

The presence of community-acquired infection was established according to clinical, imaging and microbiological parameters within 72 hours of ICU admission or hospital admission. For nosocomial infection, the diagnosis was based on the Centers for Disease Control criteria [23]. The infection sources were classified as lung, urinary, abdominal or pelvic, skin, vascular, and others.

### General management

A pulmonary artery catheter or a central venous catheter and an arterial line were placed in all patients. Norepinephrine was used to maintain mean arterial blood pressure higher than 65 mmHg after adequate fluid resuscitation (central venous or pulmonary artery occlusion pressure >12 mmHg) with either crystalloids or colloids. Antibiotics were guided according to institutional recommendations. The mechanical ventilation strategy and the renal replacement therapy (continuous hemofiltration preferably) were in accordance with the Surviving Sepsis Campaign [24]. All patients received deep venous thrombosis prophylaxis and stress ulcer prophylaxis. Continuous infusion of midazolam and fentanyl were given when necessary. Hydrocortisone (100 mg intravenously three times per day) therapy was not controlled and was administered at the discretion of the attending physician.

### Clinical evaluation

Patients were followed up to hospital discharge. The following variables were recorded: age, gender, admission category (trauma, medical or surgical), source of infection, and dates of hospital admission and ICU admission; the Acute Physiology and Chronic Health Evaluation II score [25] and the Simplified Acute Physiology II score [26] on the first admission day, and the Sequential Organ Failure Assessment [27] on days 1, 3, 5, 7, 10, 14, 18, 21, 24, 28, and 30 of ICU admission; the maximum Sequential Organ Failure Assessment score during the ICU stay [28]; the use of norepinephrine during the first five days of hydrocortisone treatment or after the corticotropin test when steroid was not given; and the hospital, ICU, and 28-day mortality rates.

### Corticotropin test and laboratory variables

All patients were submitted to a low-dose (1 μg) and a high-dose (249 μg) corticotropin test (Synacthene; Novartis Pharma Stein AG, Stein, Switzerland) within the septic shock period, and analysis of serum cortisol was performed by radioimmunoassay (Immulite; DPC Diagnostic Products, Los Angeles, CA, USA). After measurement of the serum baseline cortisol, the patients were sequentially submitted to 1 μg and 249 μg corticotropin tests with a 60-minute interval between doses. Post-stimuli cortisol levels were drawn 60 minutes after each test (cortisol 60 and cortisol 120). The cortisol 60 and cortisol 120 values minus the baseline cortisol value were called Δmax^1 ^and Δmax^249^, respectively. Cortisol results were available within 24 hours after testing. All patients were on norepinephrine at the time of the test. Laboratorial adrenal failure was defined as Δmax^249 ^≤ 9 μg/dl or baseline cortisol ≤ 10 μg/dl. Clinical adrenal failure was defined as removal of norepinephrine up to 120 hours after hydrocortisone treatment. The different baseline cortisol cutoff values referred to as adrenal failure criteria in other studies (≤15, 20, 25 and 34 μg/dl), as well as Δmax^1 ^≤ 9 μg/dl and Δmax^249 ^≤ 9 μg/dl, were analyzed concerning serum albumin levels ≤2.5 g/dl and >2.5 g/dl.

At the onset of septic shock, blood and cultures from the site of infection were obtained. The white blood cell count, the total serum protein and albumin, and the C-reactive protein level were measured on the corticotropin test day.

### Statistical analysis

Statistical analyses were conducted using SPSS 10.0 statistical package software (SPSS Incorporation, Chicago, IL, USA). The results of continuous variables are expressed as the median and interquartile range (25–75%). Nonparametric tests were used (Mann–Whitney *U *test for two independent samples, and Kruskal–Wallis *H *test for several independent samples) after exclusion of a normal distribution of the data by the Kolmogorov–Smirnov test. The chi-square test or Fisher's exact test was applied for categorical variables when indicated. Norepinephrine removal was estimated by the Kaplan–Meier method and the results were compared between groups with the log-rank test.

The baseline cortisol concentration, the most accurate predictor of the hemodynamic response to corticosteroid treatment, was determined by the area under the receiver operating characteristic curve. Pearson or Spearman's correlation coefficients correlated continuous variables. The sensitivity and specificity of each baseline cortisol cutoff value (≤15, 20, 25 and 34 μg/dl) and Δmax^1 ^were determined using a concentration of Δmax^249 ^≤ 9 μg/dl as the reference method. Differences were considered significant at *P *< 0.05.

## Results

### Patient characteristics

One hundred and two patients were included (59 female), with a median age of 74 (62–82) years. The demographic and epidemiological data are presented in Table 1. The main types of ICU admission were medical (*n *= 50) and postoperative (*n *= 47). Acute respiratory distress syndrome was diagnosed in 52 patients (51%), and the lung was the most frequent source of infection. Despite the clinical septic shock diagnosis, only 69 patients (67.6%) yielded positive microbiological results, with Gram-negative bacillus predominance. Bacteremia was detected in 22 (21.6%) patients, and nosocomial infections were as frequent as community infections. All patients were receiving norepinephrine when the corticotropin test was performed, and hydrocortisone was given to 71 patients during the shock phase for a median of 9 (5–13) days. The 28-day, ICU, and hospital mortality rates were 31.4%, 35.3%, and 45.1%, respectively.

### Laboratorial evaluation of adrenal function

The median values of the baseline cortisol level, cortisol 60, cortisol 120, Δmax^1^, and Δmax^249 ^were 17.2 μg/dl, 25.4 μg/dl, 35.5 μg/dl, 8.2 μg/dl, and 16.6 μg/dl, respectively. The median time for the corticotropin test was 2 (1–4) days after ICU admission (Table 1). AF was identified in 23 patients (22.5%) considering either Δmax^249 ^≤ 9 μg/dl or baseline cortisol ≤ 10 μg/dl, but only four patients (3.9%) presented both criteria.

There was a weak but a significant correlation between the serum albumin and baseline cortisol values – the lower the albumin levels, the lower the baseline cortisol value (*R*^2 ^= 0.1, *P *= 0.02). The same finding was observed for cortisol 60 (*R*^2 ^= 0.15, *P *= 0.002) and for cortisol 120 (*R*^2 ^= 0.1, *P *= 0.01). Nonetheless, there was no correlation between serum albumin and Δmax^249 ^values (*R*^2 ^= 0.02, *P *= 0.08). AF was identified more frequently in hypoalbuminemic patients than in those with near-normal serum albumin levels when considering the baseline cortisol value as the diagnostic criterion, especially for the cortisol cutoff value ≤ 25 μg/dl (*P *= 0.05). On the other hand, the incidence of AF according to Δmax^1 ^≤ 9 μg/dl and, mainly, Δmax^249 ^≤ 9 μg/dl criteria was not affected by serum albumin levels (Table 2).

**Figure 1:**
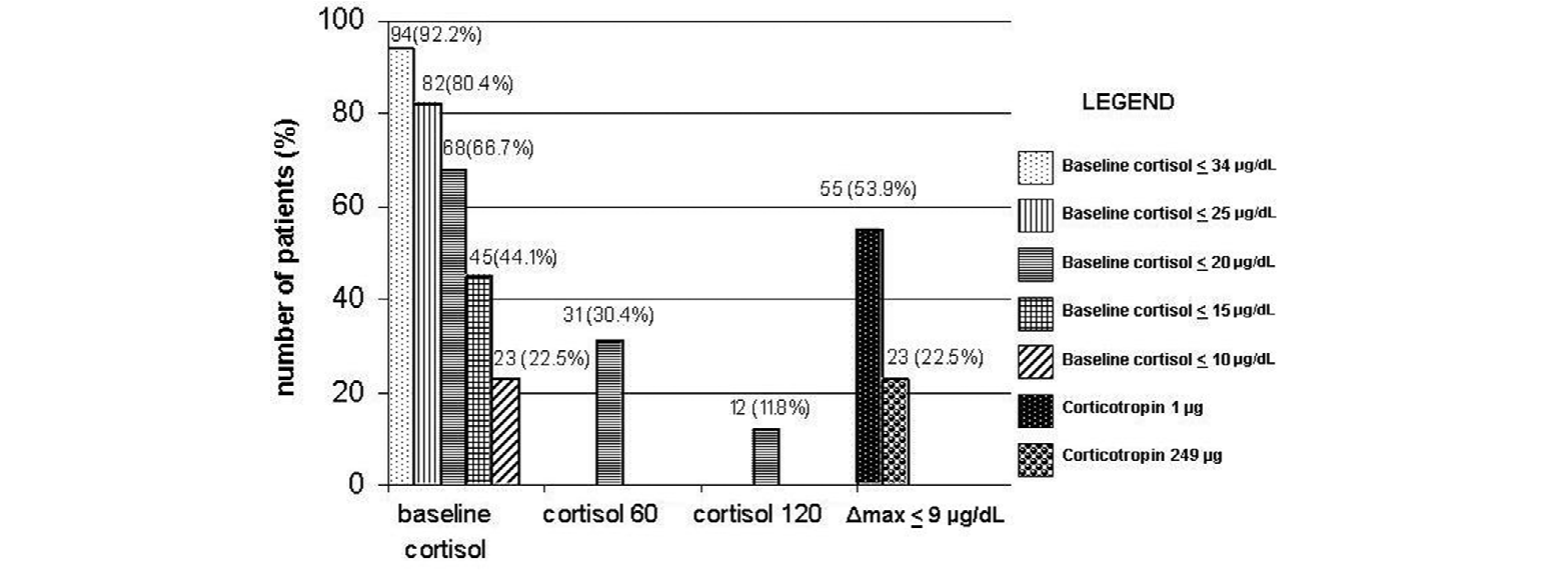
Adrenal failure diagnosis according to different criteria. The number of patients and the frequency of adrenal failure (AF) according to several baseline cortisol cutoff values (≤10, ≤15, ≤20, ≤25, and ≤34 μg/dl), the classical ambulatory criteria considering serum cortisol ≤ 20 μg/dl after stimulus with corticotropin at low dose (cortisol 60) and at high dose (cortisol 120), and the presence of Δmax^1 ^≤ 9 μg/dl and Δmax^249 ^≤ 9 μg/dl. It is observed that the higher the baseline cortisol level, the higher the theoretical diagnosis of AF. The low-dose test classified more patients with AF than the high-dose test when we considered the classical criteria of total cortisol ≤ 20 μg/dl after stimulus. This same pattern was observed for Δmax after low (Δmax^1 ^≤ 9 μg/dl) and high (Δmax^249 ^≤ 9 μg/dl) corticotropin doses.

The use of different baseline cortisol cutoff values would have shown different AF frequencies according to different cutoff values if they were considered the only criteria, where the higher the cortisol threshold, the higher the AF incidence. AF defined by the baseline cortisol level was highly variable (22.5–92.2%), which was contrary to that determined by the traditional criteria of cortisol < 20 μg/dl after corticotropin stimulus with a low does or a high dose (Figure 1).

The absolute values of Δmax^1 ^and Δmax^249 ^were well correlated (*R*^2 ^= 0.42, *P *= 0.00001). Δmax^1 ^≤ 9 μg/dl was the best criterion to predict the presence of Δmax^249 ^≤ 9 μg/dl, presenting an odds ratio of 2.29 (95% confidence interval, 1.74–3; *R *= 0.45, *P *< 0.00001).

### Clinical evaluation of adrenal function

**Figure 2:**
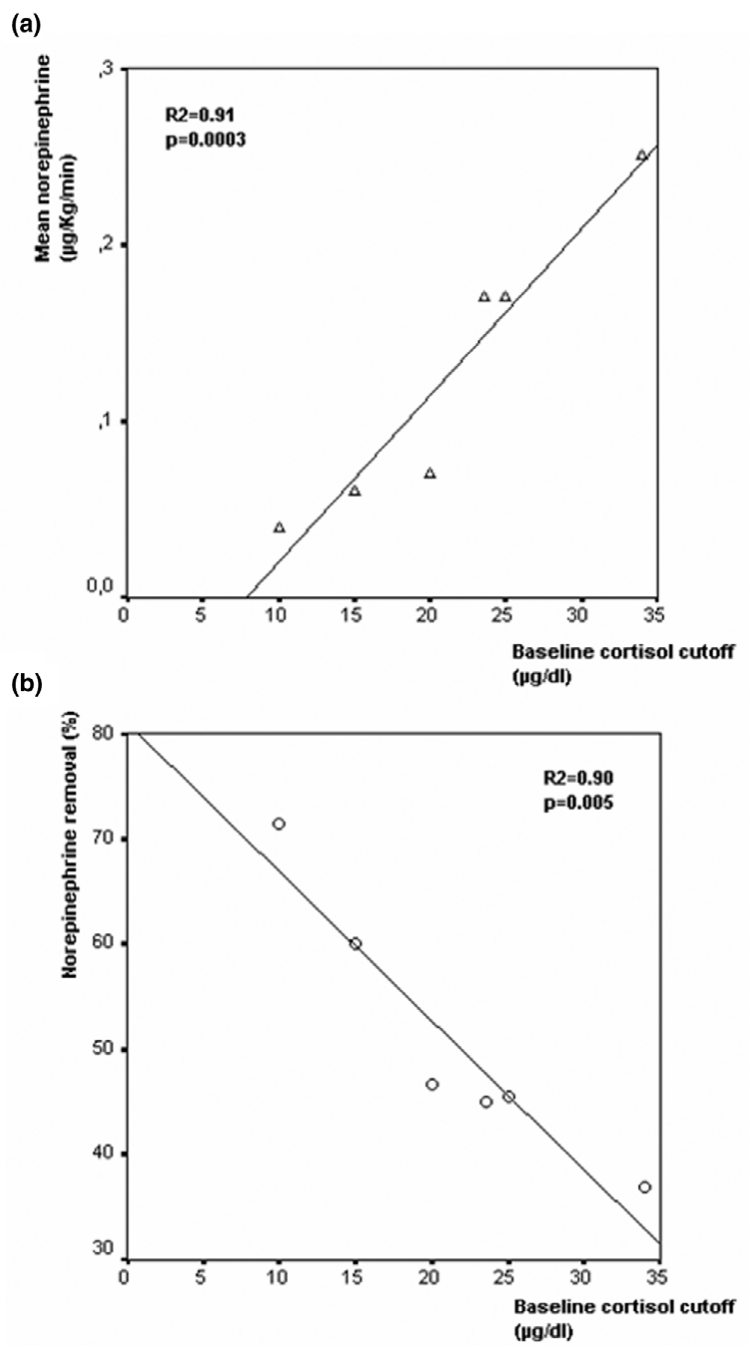
Different baseline cortisol cutoff values and vasopressor dependence. **(a) **The mean norepinephrine dose and **(b) **the probability of norepinephrine removal during the five days after the corticotropin test in patients not treated with hydrocortisone.

The dose of norepinephrine was higher in patients treated with hydrocortisone than in nontreated patients on the day before beginning steroid treatment (0.35 μg/kg/minute versus 0.13 μg/kg/minute, *P *= 0.03). It was impossible to determine a baseline cortisol value, as well as Δmax^1 ^and Δmax^249 ^values, which would best define norepinephrine removal in both the general population and in the group of patients treated with hydrocortisone. Nonetheless, in the nontreated group the area under the receiver operating characteristic curve of baseline cortisol was 0.81, and the cortisol value of 23.6 μg/dl was associated with the highest sensitivity (0.83) and the highest specificity (0.75) for norepinephrine removal during the first five days after the corticotropin test. As a general finding, in the nontreated group we observed that the higher the baseline cortisol, the higher the mean dose of norepinephrine and the lower the probability of norepinephrine weaning (Figure 2).

## Discussion

In the present study we analyzed the diagnosis of AF in a heterogeneous group of patients with septic shock. AF failure was identified more reliably by Δmax^249 ^≤ 9 μg/dl than by several baseline cortisol cutoff values since serum albumin levels directly affected the measurement of the serum total cortisol at baseline and after low and high doses of corticotropin, as well as the frequency of different baseline total cortisol cutoff values; however, serum albumin levels did not affect the measurement of Δmax levels and the frequency of Δmax^249 ^≤ 9 μg/dl. Additionally, in the group of patients not treated with hydrocortisone, the baseline serum total cortisol level was a prognostic marker for vasopressor dependence – the higher the baseline cortisol, the higher the norepinephrine requirement.

Serum albumin levels influenced cortisol measurement at baseline and after low and high corticotropin doses. We observed that the lower the serum albumin, the higher the incidence of AF when considering the baseline cortisol cutoff value as the diagnostic criterion, especially for the value of 25 μg/dl. In the context of reduced serum albumin levels, therefore, the incidence of AF could be overestimated. On the other hand, this effect was not observed for Δmax^1 ^and Δmax^249 ^values, and AF diagnosis regarding Δmax^249 ^≤ 9 μg/dl was not influenced by serum albumin levels. These findings highlight the importance of measuring the adrenal gland reserve (Δmax) and confirm the results of previous studies that used Δmax^249 ^≤ 9 μg/dl as the diagnostic criterion for AF in critically ill patients and hypoproteinemic patients [9,10,16,17].

Our data are in accordance with those of Hamrahian and colleagues [20], which also showed significant variation of the baseline total cortisol level, but not of the free cortisol fraction, in patients with near-normal and low serum albumin – suggesting that measurement of free cortisol could be a more reliable method than measurement of total cortisol for diagnosis of AF in critical illness. We were unable to measure the free cortisol level in the present work but we did find, similar to free cortisol, that Δmax^249 ^≤ 9 μg/dl was not affected by serum albumin, allowing it to be a diagnostic option in the setting of hypoproteinemia, where baseline total cortisol is susceptible to serum albumin variations and free cortisol measurement is not available.

There are some points to be considered favoring Δmax^249 ^≤ 9 μg/dl in comparison with the free cortisol dosage. First, Δmax^249 ^≤ 9 μg/dl is easier to measure and produces faster results with lower costs and more widespread availability in the ICU compared with free cortisol measurement. Second, although baseline total cortisol levels may overestimate AF in comparison with the free cortisol fraction dosage, the measurement of free cortisol is still not validated for the prognosis and as a guide for diagnosis and treatment of hypothalamic–pituitary–adrenal axis dysfunction in critically ill septic patients. In a very recent review in the literature about current problems when evaluating hypothalamic–pituitary–adrenal function, Arafah [21] reported significant Δmax ≤ 9 μg/dl variation according to serum albumin. This result was different from that found in our study, and we can speculate that differences in the studied population and the time for performing the corticotropin test might explain the distinct findings.

Several studies have shown the prognostic role of Δmax^249 ^≤ 9 μg/dl as it enables the identification of a specific group of patients with low adrenal reserve, also known as 'relative adrenal failure', who could benefit from steroid replacement [5,9,10,17,29]. Annane and colleagues [9] demonstrated that patients with septic shock nonresponsive to 250 μg corticotropin (Δmax ≤ 9 μg/dl) had a greater dependence on vasopressor therapy and a higher hospital mortality rate than those responsive to corticotropin stimulation (Δmax > 9 μg/dl), and that a seven day treatment combination of hydrocortisone and fludrocortisone significantly reduced vasopressor dependence and hospital risk of death. The same benefit of steroid treatment, however, was not observed in the responders to the corticotropin test (Δmax > 9 μg/dl). Besides the reliability of Δmax^249 ^≤ 9 μg/dl for the diagnosis of adrenal insufficiency, the measurement of Δmax^249 ^also enabled the identification of septic shock patients at a high risk of death who should receive hydrocortisone, avoiding excessive use of steroids.

Alongside the problem concerning the direct effect of serum albumin variation on total cortisol levels and consequently AF diagnosis, the definition of AF based on baseline total cortisol levels may be extremely variable according to the threshold established. We found that the higher the cutoff value used, the higher the incidence of adrenal insufficiency. This problem was well explored by Bernard and colleagues [30], who tested different criteria of adrenal insufficiency in a population of patients with recent traumatic brain injury. They showed that the incidence of adrenal insufficiency varied from 22% to 100% according to the diagnostic criteria, which was very similar to our findings. In an attempt to solve the problem, Arafah [21] correlated the levels of baseline serum total cortisol and free cortisol in critically ill patients with low and near-normal serum albumin. The author proposed that in hypoalbuminemic subjects the normal total cortisol level should be ≥9.5 μg/dl at baseline and ≥15.5 μg/dl after the cosyntropin test. These values corresponded to free cortisol concentrations of 1.8 μg/dl and 3.1 μg/dl, respectively, which is considered the normal cortisol range in critical illness by the author. It seems that such an approach is more appropriate and rational than simply trying to establish a fixed diagnostic cutoff value that neglects serum protein variations.

AF was detected in 23 patients (22.5%) when considering a baseline cortisol level ≤ 10 μg/dl or Δmax^249 ^≤ 9 μg/dl. Only four of these patients presented both criteria. This is in accordance with the majority of the studies that have reported a wide range of incidence varying from 0 to 77% depending on the studied population and adrenal insufficiency diagnostic criteria [5,10,11,13,20,21,31-34].

In the more homogeneous group of patients who did not receive hydrocortisone replacement, it was possible to correlate the baseline serum total cortisol levels with vasopressor dependence. Higher norepinephrine doses and greater norepinephrine dependence were observed in patients with higher baseline cortisol levels. The baseline cortisol value of 23.6 μg/dl, which best discriminated individuals who were weaned from norepinephrine, was very close to the value of 23.7 μg/dl described by Marik and Zaloga [13]. Other authors have also shown bad prognosis with increased baseline cortisol, correlating the highest cortisol levels with the most severe illness and the highest risk of mortality [5,13,35]. Since in our study hydrocortisone therapy was not controlled, patient selection bias might have occurred explaining why it was not possible to determine a value of baseline cortisol, Δmax^1^, or Δmax^249 ^that could predict norepinephrine removal either in the general population of the study or in the steroid patient group. Additionally, other problems related to the study design, such as the relatively small sample size and the long period of the study, could have contributed to difficult outcome analysis.

## Conclusion

Our study showed that the incidence of AF was 22.5% in a heterogeneous group of septic shock patients and that Δmax^249 ^≤ 9 μg/dl may be a better option for AF diagnosis whenever free cortisol measurement is not available, since Δmax^249 ^≤ 9 μg/dl was not affected by serum albumin while the baseline total cortisol levels and their cutoff values varied directly according to albumin levels. As a potential result, the use of the baseline total cortisol level for AF diagnosis could lead to misleading classification and excessive steroid replacement in the context of hypoproteinemia. In the group of patients without hydrocortisone therapy, we found that a baseline cortisol level ≤ 23.6 μg/dl was the best value to predict norepinephrine removal.

## Key messages

• Δmax^249 ^≤ 9 μg/dl may be a better option for AF diagnosis whenever free cortisol measurement is not available, since Δmax^249 ^≤ 9 μg/dl was not affected by serum albumin while the baseline total cortisol level varied directly according to albumin levels.

• The use of baseline total cortisol for AF diagnosis could lead to misleading classification and excessive steroid replacement in the context of hypoproteinemia.

• In the group of patients without hydrocortisone treatment, a baseline serum total cortisol level higher than 23.6 μg/dl was correlated with greater vasopressor dependence.

## Abbreviations

AF = adrenal failure; Δmax = difference between the highest serum cortisol value after corticotropin stimulus and the baseline cortisol value; Δmax^1^= difference between serum cortisol value after low (1 μg) corticotropin dose and the baseline cortisol value; Δmax^249^= difference between serum cortisol value after high (249 μg) corticotropin dose and the baseline cortisol value; ICU = intensive care unit.

## Competing interests

The authors declare that they have no competing interests.

## Authors' contributions

DRS conceived the design of the study, was responsible for collection of data, participated in the statistical analysis and interpretation of data, and assisted in drafting the manuscript. JCRV participated in the conception and design of the study and helped to draft the manuscript, revising it critically for important intellectual content. JRR participated in the conception and design of the study, and in the statistical analysis, and helped to draft the manuscript, revising it critically for important intellectual content.

## Figures and Tables

**Table 1:** Clinical, biological and infection data of patients

Sex	
Female	59 (57.8)
Male	43 (42.2)
Age (years)	74 (62–82)
Admission diagnoses	
Postoperative	47 (46.1)
Medical	50 (49)
Trauma	5 (4.9)
Acute respiratory distress syndrome	52 (51)
Length of intensive care unit stay (days)	18 (12–27.3)
Length of hospital stay (days)	33 (19–85.3)
Days for corticotropin test^a^	2 (1–4)
Total serum protein (g/dl)^b^	4.7 (4.1–5.2)
Serum albumin (g/dl)^b^	2 (1.7–2.4)
White blood cells (cells/μl)	11,550 (8,275–16,500)
C-reactive protein (mg/dl)	16.3 (10.5–25)
Baseline cortisol levels (μg/dl)	17.2 (10.6–23.8)
Cortisol 60 (μg/dl)	25.4 (18.6–39.2)
Cortisol 120 (μg/dl)	35.5 (24.5–49.9)
Δmax^1 ^(μg/dl)	8.2 (3.5–17.3)
Δmax^249 ^(μg/dl)	16.6 (10–26)
Mean daily dose of norepinephrine^c ^(μg/kg/minute)	0.13 (0.04–0.54)
Sequential Organ Failure Assessment score (corticotropin test day)	9 (7.8–11)
Maximum Sequential Organ Failure Assessment score	10 (9–13)
First-day Sequential Organ Failure Assessment score	7 (4–10)
Acute Physiologic and Chronic Heath Evaluation II score	20 (17–23)
Simplified Acute Physiology II score	43.5 (36–52.3)
28-day mortality rate	32 (31.4)
Intensive care unit mortality rate	36 (35.3)
Hospital mortality rate	46 (45.1)
Site of infection	
◦Intra-abdominal	28 (27.5)
◦Soft tissue abscess/cellulitis	3 (2.9)
◦Lung	58 (56.9)
◦Central nervous system	1 (1)
◦Urinary tract	10 (9.8)
◦Endovascular infection	2 (2)
Microorganisms^d^	
◦Gram-negative bacillus	72 (61.6)
◦Gram-positive coccus	39 (33.3)
◦Fungus	6 (5.1)
Bacteremia	22 (21.6)
Type of infection	
◦Community	51 (50)
◦Nosocomial	51 (50)

Data presented as *n *(%) or median (interquartile range). Cortisol units: 1 μg/dl = 27.6 nmol/l. Cortisol 60, serum cortisol level drawn 60 minutes after stimulus with 1 μg corticotropin; Cortisol 120, serum cortisol level drawn 60 minutes after stimulus with 249 μg corticotropin (120 minutes after serum baseline cortisol); Δmax^1^, cortisol 60 value minus the baseline cortisol level; Δmax^249^, cortisol 120 value minus the baseline cortisol level. ^a^Difference between corticotropin test day and intensive care unit admission day. ^b^Protein and albumin levels were not measured in 15 patients. ^c^Mean daily dose of norepinephrine during the first five days after hydrocortisone usage or after the corticotropin test. ^d^Microbiological identification was obtained in 69 patients (67.6%).

**Table 2:** Adrenal function according to the serum albumin level

	Albumin ≤ 2.5 g/dl (*n *= 75)	Albumin > 2.5 g/dl (*n *= 12)	*P *value
Baseline cortisol (μg/dl)	15.5 (10.4–23.4)	22.4 (15.5–26.7)	0.04*
Cortisol 60 (μg/dl)	23.5 (17.2–36.6)	40.3 (27–51.5)	0.009*
Cortisol 120 (μg/dl)	30.4 (23.2–46.7)	41.9 (33.1–54.9)	0.04*
Δmax^1 ^(μg/dl)	6.9 (3.5–13.2)	13.2 (3.7–26.6)	0.24
Δmax^249 ^(μg/dl)	14.5 (9.4–23.4)	18.8 (9.9–28.9)	0.48
Baseline cortisol ≤ 10	17 (22.7)	1 (8.3)	0.45
Baseline cortisol ≤ 15	37 (49.3)	3 (25)	0.12
Baseline cortisol ≤ 20	52 (69.3)	6 (50)	0.2
Baseline cortisol ≤ 23.6	58 (77.3)	6 (50)	0.07
Baseline cortisol ≤ 25	63 (84)	7 (58.3)	0.05*
Baseline cortisol ≤ 34	70 (93.3)	11 (91.7)	1.0
Δmax^249 ^≤ 9	18 (24)	3 (25)	1.0
Δmax^1 ^≤ 9	46 (61.3)	4 (33.3)	0.07

Data presented as *n *(%) or median (interquartile range). Protein and albumin levels were not measured in 15 patients. Cortisol units: 1 μg/dl = 27.6 nmol/l. Cortisol 60, serum cortisol level drawn 60 minutes after stimulus with 1 μg corticotropin; Cortisol 120, serum cortisol level drawn 60 minutes after stimulus with 249 μg corticotropin (120 minutes after serum baseline cortisol); Δmax^1^, cortisol 60 value minus the baseline cortisol level; Δmax^249^, cortisol 120 value minus the baseline cortisol level.
